# Kestrel-Prey Dynamic in a Mediterranean Region: The Effect of Generalist Predation and Climatic Factors

**DOI:** 10.1371/journal.pone.0004311

**Published:** 2009-02-23

**Authors:** Juan A. Fargallo, Jesús Martínez-Padilla, Javier Viñuela, Guillermo Blanco, Ignasi Torre, Pablo Vergara, Liesbeth De Neve

**Affiliations:** 1 Departamento de Ecología Evolutiva, Museo Nacional de Ciencias Naturales-CSIC, Madrid, Spain; 2 Aberdeen Centre for Environmental Sustainability (ACES), University of Aberdeen & The Macaulay Institute, School of Biological Sciences, Aberdeen, United Kingdom; 3 Instituto de Investigación en Recursos Cinegéticos (CSIC-UCLM), Ciudad Real, Spain; 4 Museu de Granollers-Ciències Naturals, Barcelona, Spain; 5 Departamento de Biología Animal y Ecología, Facultad de Ciencias, Universidad de Granada, Granada, Spain; University of California, Berkeley, United States of America

## Abstract

**Background:**

Most hypotheses on population limitation of small mammals and their predators come from studies carried out in northern latitudes, mainly in boreal ecosystems. In such regions, many predators specialize on voles and predator-prey systems are simpler compared to southern ecosystems where predator communities are made up mostly of generalists and predator-prey systems are more complex. Determining food limitation in generalist predators is difficult due to their capacity to switch to alternative prey when the basic prey becomes scarce.

**Methodology:**

We monitored the population density of a generalist raptor, the Eurasian kestrel *Falco tinnunculus* over 15 years in a mountainous Mediterranean area. In addition, we have recorded over 11 years the inter-annual variation in the abundance of two main prey species of kestrels, the common vole *Microtus arvalis* and the eyed lizard *Lacerta lepida* and a third species scarcely represented in kestrel diet, the great white-toothed shrew *Crocidura russula*. We estimated the per capita growth rate (PCGR) to analyse population dynamics of kestrel and predator species.

**Principal Findings:**

Multimodel inference determined that the PCGR of kestrels was better explained by a model containing the population density of only one prey species (the common vole) than a model using a combination of the densities of the three prey species. The PCGR of voles was explained by kestrel abundance in combination with annual rainfall and mean annual temperature. In the case of shrews, growth rate was also affected by kestrel abundance and temperature. Finally, we did not find any correlation between kestrel and lizard abundances.

**Significance:**

Our study showed for the first time vertebrate predator-prey relationships at southern latitudes and determined that only one prey species has the capacity to modulate population dynamics of generalist predators and reveals the importance of climatic factors in the dynamics of micromammal species and lizards in the Mediterranean region.

## Introduction

The study of demographic patterns in animal populations is a basic, as well as puzzling, research subject, important from a purely scientific perspective, up to conservation as well as from management points of view. A general conviction shared by ecologists is that trophic interactions (plant-herbivore, predator-prey, or host-parasitoid) are key factors affecting temporal oscillations (regular or irregular) of population numbers [1]–[3]. Population dynamics may also be affected by endogenous density-dependent processes based on interactions among individuals within a population or interactions between populations of two or more different species [4]–[6]. Long time-series data are essential to investigate the role of endogenous and exogenous parameters affecting population fluctuations. A vast majority of studies in this field, particularly in rodents, has been carried out in northern holarctic ecosystems, from where most hypotheses have been posited [2], [3], [7]–[11]. Consequently, analyses based on time-series data gathered in areas other than northern latitudes are currently strongly needed to broaden the spectrum of knowledge about parameters affecting population dynamics and to provide information about the effects of environmental stochasticity.

Small mammals and their predators, are among the most studied species and systems. The regular inter-annual fluctuations or cycles detected in many of rodent populations have been the subject of a great number of studies developing hypotheses about this striking phenomenon [2], [7], [8]–[10], [12]–[18].; It seems that cyclic population dynamics, mainly in microtine species, are observed more often at high than at low latitudes [8]–[10], [13], [14], [19], [20]. Although differences between high and low latitude rodent population dynamics could be due to a variety of intra-population processes [19], [21], regular cyclic oscillations at high latitudes have often been explained as a result of second-order negative feedback (slow and delayed density dependence) caused by interactions with specialist predators, while more stable or non-cyclic fluctuating rodent populations in southern latitudes would be a result of first-order feedback (rapid and direct density dependence) because of regulation by generalist predators [8], [10], [14], [20], [22]–[26 but see 13,15,27,28], considering “order” of a dynamic system as the number of variables involved in the endogenous structure, or the maximum time lag in the dynamic [1].

The change in the type of predator from more specialized in the north to more generalist in the south may not be strictly due to a change in the predator community, but also to a change in predator behaviours. For example, the Eurasian kestrel *Falco tinnunculus* is considered a nomadic rodent-specialist predator in northern Europe [24], [29], [30] but a nomadic and/or resident generalist predator in the south [31]–[33]. This could imply, in the case of kestrels, a possible change in population dynamic of their prey derived from trophic interactions [24]. It is, thus, obvious the necessity of studying inter-annual fluctuations of single organisms and predator-prey interactions in different regions, as emphasized by many authors [3], [7], [17], [18], [24], [28].

To our knowledge, no time series of small-mammal abundances have been described in the southern Mediterranean area of Europe. In the case of avian predators, the role of trophic interactions or food-limitation has mainly been documented in vole-specialists [34]. In contrast, little is known about food limitation in vertebrate generalist foragers [35].

In this study, we analyse a demographic time-series in a Mediterranean predator-prey system. We first describe inter-annual population numbers of Eurasian kestrels for a 15-year period. Next we describe the population dynamics of three kestrel prey species over 11-year period, including prey occupying different ecological niches: the insectivorous eyed lizard *Lacerta lepida*, the herbivorous common vole *Microtus arvalis* and insectivorous white-toothed shrew *Crocidura russula*. We analyse the feedback structure (intrinsic processes) and exogenous factors (climate) determining population dynamics in kestrel and prey species by analysing the per capita changes in population abundances.

## Methods

### Study area

The study was performed in the Campo Azálvaro region, a highland grassland of central Spain (40°40′N, 4°20′W). The area is a treeless flat valley at 1300 m a.s.l. located between Malagón and Ojos Albos mountain ridges and devoted mainly to cattle raising [36], [37]. The climate of this region is humid Mediterranean, with dry and warm summers (mean temperature from June to August  = 19°C and mean monthly precipitation  = 22.9 mm) and cold winters (mean temperature from December to February  = 3.7°C). Climate data were provided by the Regional Center of Meteorology from Castilla y León. Total precipitation and total number of days with snow cover for the entire study period were obtained monthly from two close meteorological stations in the area (El Espinar and San Rafael). We considered mean values from both stations. During the last 15 years, mean annual rainfall was 705±180 ranging from 475 to 1071 mm, mean annual temperature was 10.7±0.4°C ranging from 9.9 to 11.6°C and mean number of days with snow cover were 30.5±8.9, ranging from 22 to 51 days.

### Kestrel population

**Figure 1:**
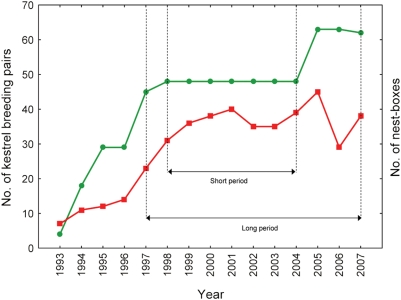
Kestrel and nest-box numbers. Inter-annual variation in the number of nest-boxes installed (green line and dots) and the number of Eurasian kestrel pairs breeding (red line and squares) in the study area. The 7-year period in which the number of nest-boxes was constant (short period) and the 11-year period (long period) of trapping prey species were indicated.

Yearly censuses of Eurasian kestrels in the area were made from 1993 to 2007. During this period, nest-boxes for kestrel breeding were erected promoting a remarkable increase in the breeding population of this raptor [36]. In 1993, the study area had only four nest-boxes that had been erected in 1988. Fourteen new nest-boxes were installed in the winter of 1993–94, 11 more in 1994–95, 16 more in 1996–97, three more in early spring 1998 and 15 more in February 2005. In the winter of 2007 a tree with a nest-box had fallen. A total of 62 nest-boxes were finally set in an area of 22 km^2^ (Fig. 1). All breeding pairs nesting in nest-boxes or in other nesting sites in this area were recorded. Common voles and eyed lizards represent 1.8% and 1.8% of the prey items consumed by kestrels and 7% and 19% of the biomass, respectively. Great white-toothed shrews represent 0.1% of the prey consumed and 0.1% of the biomass (data collected in spring from 1995 to 1998 [31 and unpublished data]). A fraction of this kestrel population is resident, staying in the area over the entire year [31], however there is also a migrating fraction in our population (data from the Spanish Bird Migration Center).

### Trapping

**Figure 2:**
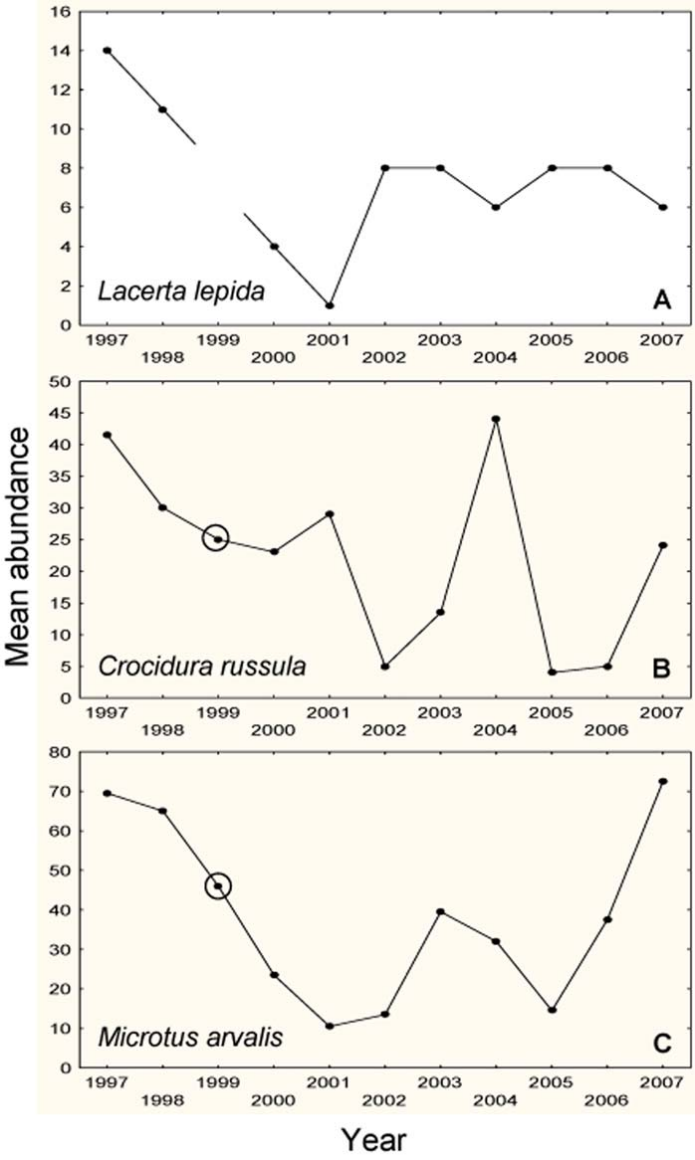
Inter-annual fluctuation in the abundance of prey species. Inter-annual variation in the abundance of trapped eyed lizards (A), white-toothed shrews (B) and common voles (C). Black dots represent mean annual values (summer+autumn)/2. Dots inside a circle represent only autumn values. Eyed lizard abundances correspond to summer trappings.

The abundance of eyed lizards, common voles and great white-toothed shrews (Fig. 2) was assessed by two trapping bouts per year from 1997 to 2007. Every year a trapping was done in June (summer session) and a second late in October (autumn session). Due to logistic problems, in 1999 we only carried out one trapping session in autumn. Eyed lizards were only trapped in the summer session since low air temperature during autumn in our study area prevents lizard activity. Summer trapping was always carried out on sunny days and rainy or snowy weather was also avoided during autumn trapping. Enclosures and roadsides constitute the optimal habitats for small mammal communities in our study area [37], [38]. One hundred live Sherman traps were placed in four plots (25 each) during new moon periods to avoid effects of moonlight on small mammal activity [37]. Two trapping plots were in roadsides and the other two in both enclosures. In the roadside plots, traps were placed in two parallel lines of 12 and 13 traps each on both sides of the road. In enclosure plots, traps were placed in five parallel lines of five traps each. All trapping areas were located more than one km apart.

We baited the traps with a mixture of tuna, flour and oil and with a piece of apple. During the autumn session, traps were supplied with cotton bedding, to reduce the effect of cold weather. Traps were set under the cover of herb and were kept operatives for four consecutive days. We placed them at midday on the first day and removed them at sunrise on the fourth day. We then made six trap revisions over the four day period: three at sunset and three at sunrise. Small mammals were marked by haircutting to avoid counting repeated captures. We used counts (number of different individuals trapped within the four days [39]) as estimates of population size in each study plot, assuming that the unseen proportion of the population is constant [40] and that in some situations, counts and estimates yielded similar results [41].

### Analytical procedures

We used the total number of animals trapped during all six trapping bouts as an index of small mammal abundance per season for each species separately. The yearly abundance of trapped species was estimated as the mean value of both trapping bouts of each year (summer and autumn). For 1999 only the abundance for autumn was given.

Estimates of the roles of density dependence and exogenous factors (precipitation, temperature and nest-site availability) on the per capita growth rate log_e(N_t/N_t-1) were done by fitting different models of the form:
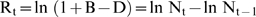
(1)where 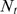 is the density of population 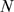 (trapped species or kestrels) at time *t*, B and D per capita birth and death rates, respectively and *R_t_* is the realized logarithmic per capita growth rate (PCGR) or rate of change of the population of the time interval. Rainfall or water in the broader sense is a surrogate for primary productivity [42], [43] whose effects are well-known in arid and semi-arid ecosystems increasing vegetation cover, seeds, insects and consequently small mammals and lizard consumers [17], [18], [44]–[46] for which rainfall can be incorporated in the models in place of food resources [17], [45]. On the basis of equation 1 we constructed new models integrating the feedback structure, predation forces (in the case of prey species), food abundance (prey density in the case of kestrels and rainfall in the case of trapped species), ambient temperature implying a linear regression of R_t_ on each new term included in the model as derived from Lotka-Volterra equations in the logarithmic Gompertz version [17], [47], [48]. Gompertz approach has been commonly used to relate linearly PCGR to the logarithm of lagged densities and climatic factors [17], [47], [48].

In the case of prey species:
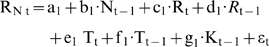
(2)where a_1_, b_1_, c_1_, d_1_, e_1_, f_1_ and g_1_ are constant parameters estimated by multiple linear regression, N_t−1_ is one-year lagged population densities of prey species, R is rainfall, T is temperature and K_t−1_ is one-year lagged density of kestrels. The term *ε_t_*,is the noise term, normally being distributed N (0, σ). All terms are log transformed.

In the case of kestrels:
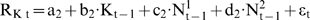
(3)where a_2_, b_2_, c_2_ and d_2_ are constant parameters estimated by multiple linear regression, and N^1^, N^2^ are lagged population densities of prey species. In addition, once we knew what prey species showed significant correlation with kestrel PCGR, we also included the sum of prey species as independent variables [48], [49] to evaluate the increase in the variance explained by the model:
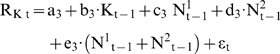
(4)


Finally, an alternative approach to modelling trophic interactions is to relate the PCGR to the ratio of consumers to their food resources, these models being known as logistic food webs [48]–[50].

In the case of prey species:
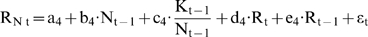
(5)Where once again a_4_, b_4_, c_4_, d_4_ and e_4_ are constant parameters estimated by multiple linear regression. In this model the terms were a combination of the ratio of trapped species and their food resources (winter rainfall) in the way N_t−1_/Rain_t_ or N_t−1_/Rain_t−1_ [45].

In the case of kestrels:
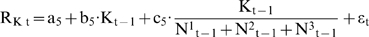
(6)Where a_5_, b_5_ and c_5_ are again constant parameters estimated by multiple linear regression.

We used corrected the Akaike's information criterion corrected for small sample size (AICc) [51] to select the best model for each species, with smaller values indicating a more parsimonious model. It was subjectively assumed that a difference of less than two units in AICc values is not significant [51].

## Results

The abundance of kestrels steadily increased during our study period, matching the pattern of nest-box provisioning since 1994 (*r* = 0.87, *F*_1,13_ = 42.74, *P*<0.0001, Fig. 1). Nest-box management from 1994 to 2007 is the only possible variable explaining the drastic increase of kestrels area since nest site availability is a limiting factor in the study area. However, full nest-box occupation was never reached, and additional provisioning of nest boxes in 2005 did not clearly increase kestrel population after 1998 (Fig. 1). The mean number of kestrels breeding from 1997 to 2004 (34.6±1.8) did not differ significantly (*t-test t*_1,9_ = 0.65, *P* = 0.56) from those breeding during the last three years (37.3±2.7). Even so, we did separate models considering first the seven-year period (“short period”: 1998–2004) during which the number of nest boxes where constant (Fig.1) and then a second set of analyses including the whole eleven-year period (“long period”: 1997–2007) from which we had prey abundance estimations (Fig. 1 and Fig. 2).

**Figure 3:**
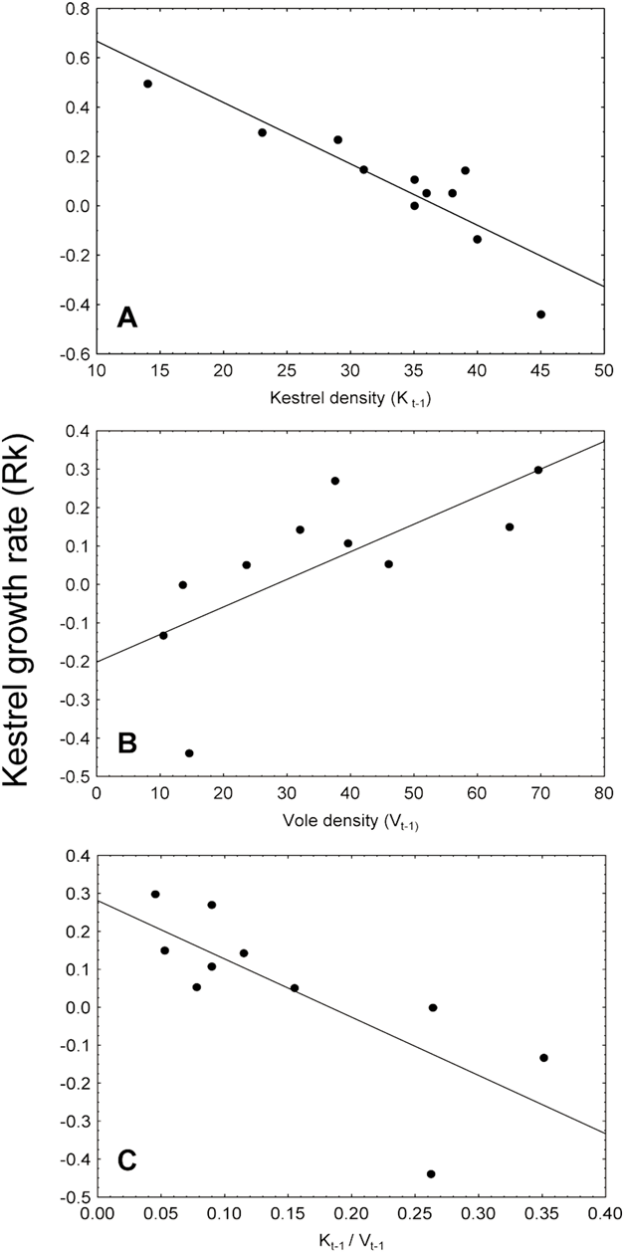
Factors affecting kestrel population dynamic. Linear relationship between the per capita growth rate of Eurasian kestrels and one-year lagged densities of kestrels (A), common voles (B). The linear relationship between the per capita growth rate of kestrels and the trophic term is also showed (C).

Kestrel results for the “short period” showed no discrimination among four best models. The model with a lower *AICc* value describing the per capita growth rate of kestrels was that built only with the self-regulation term, that is, the kestrel density of the preceding year (model 1k, Table 1). Population density alone explained 82% of the variance. The second best model (attending to *AICc* values) described kestrel growth rate as a function of the self-regulation and trophic (ratio of kestrel density to vole density) terms (12k, Table 1). The third (2k) and fourth (10k) ranked models were composed by only one term: vole density of the preceding year and the trophic kestrel/vole ratio respectively. Vole and lizard abundance explained 79% and 77% of the variation in kestrel growth rate while shrew abundance explained only 12%. Including four more years in the time series (“long period”), we obtained the same four best models found for the “short period”. In this case, the model composed of only the self-regulation term (model 15k) showed *ΔAICc*>2 (Table 1). As a whole, kestrel growth rate was negatively affected by kestrel population density and positively with the density of one of its prey (common vole; Fig. 3). Models including nest-boxes (18k and 19k) were clearly discriminated with regard to the best model (16k, Table 1). Models with parameter estimates, parameter bias and confident intervals [52] are shown in the table 2.

**Table 1:** Selected kestrel population-dynamic models.

Model	*R^2^*	*AICc*	*ΔAICc*	*P*
**Short Period (1998–2004)**				
Lotka-Volterra (Gompertz modification)				
**1k) Rk = −b (K_t−1_)**	**0.82**	**−9.23**	**0.00**	**0.005**
**2k) Rk = +c (V_t−1_)**	**0.79**	**−8.17**	**1.06**	**0.008**
3k) Rk = +d (L_t−1_)	0.77	0.07	9.30	0.021
4k) Rk = +e (S_t−1_)	0.12	1.66	10.89	0.433
5k) Rk = −b (K_t−1_)+c (V_t−1_)	0.93	−2.79	6.44	0.004
6k) Rk = −b (K_t−1_)+d (L_t−1_)	0.82	25.24	34.47	0.073
7k) Rk = −b (K_t−1_)+c (V_t−1_)+d (L_t−1_)	0.96	41.23	50.46	0.057
Logistic				
8k) Rk = −b (K_t−1_)−f [K_t−1_/(V_t−1_+L_t−1_)]	0.94	21.68	30.91	0.013
9k) Rk = −b (K_t−1_)−g [K_t−1_/(V_t−1_+L_t−1_+S_t−1_)]	0.88	26.10	35.33	0.042
**10k) Rk = −h (K_t−1_/V_t−1_)**	**0.76**	**−7.36**	**1.87**	**0.012**
11k) Rk = −g (K_t−1_/L_t−1_)	0.66	2.38	11.61	0.050
**12k) Rk = −b (K_t−1_)−h (K_t−1_/V_t−1_)**	**0.97**	**−8.48**	**0.75**	**0.001**
13k) Rk = −b (K_t−1_)−g (K_t−1_/L_t−1_)	0.92	23.62	32.85	0.022
14k) Rk = −f [K_t−1_/(V_t−1_+L_t−1_)]	0.91	−5.90	3.33	0.003
**Long Period (1997–2007)**				
Lotka-Volterra (Gompertz modification)				
15k) Rk = −b (K_t−1_)	0.82	−1.54	2.23	0.005
**16k) Rk = +c (V_t−1_)**	**0.79**	**−3.77**	**0.00**	**0.008**
**17k) Rk = b (K_t−1_)+c (V_t−1_)**	**0.71**	**−2.05**	**1.72**	**0.023**
18k) Rk = +k (B_t−1_)	0.26	1.13	4.90	0.060
19k) Rk = +c (V_t−1_)+k (B_t−1_)	0.60	−0.12	3.65	0.043
Logistic				
**20k) Rk = −b (K_t−1_)−h (K_t−1_/V_t−1_)**	**0.75**	**−3.57**	**0.20**	**0.008**

Per capita growth rate of kestrels *Falco tinnunculus* (Rk) for short (7 years) and long (11 years) periods. Log-transformed population densities of Kestrels (K), Voles *Microtus arvalis* (V), eyed lizards *Lacerta lepida* (L), white-toothed shrews *Crocidura russula* (S) are included in the models. The effect of nest boxes (B) for the “long period” is also shown. Bold type represents best models according to Akaike (AICc) criterion.

**Table 2:** Parameter estimates and confident intervals of population dynamic models.

Models	Parameter	Parameter	Parameter	Parameter	Bias
	Estimate	Estimate	Estimate	Estimate	
**Kestrels**					
Short period					
Rk =	23.706 (1.11, 3.62)	−0.6534 K_t−1_ (1.01, 0.29)			0.0067
Rk =	−0.4671 (0.80, 0.13)	+0.1580 V_t−1_ (0.06, 0.25)			0.0069
Rk =	0.2218 (0.11, 0,34)	−0.9896 [K_t−1_/V_t−1_] (1.67, 0.34)			0.0065
Rk =	16.592 (0.93, 2.38)	−0.4267 K_t−1_ (0.64, 0.21)	−0.5741 [K_t−1_/V_t−1_] (0.91, 0.24)		0.0026
Long period					
Rk =	−0.762 (1.36, 0.16)	+0.2403 V_t−1_ (0.06, 0.41)			0.1058
Rk =	19.264 (1.40, 1,60)	−0.6337 K_t−1_ (1.40, 0.14)	+0.10919 V_t−1_ (0.11, 0.33)		0.0849
Rk =	23.443 (0.13, 4.55)	−0.6128 K_t−1_ (1.26, 0.04)	−0.8200 [K_t−1_/V_t−1_] (1.92, 0.36)		0.008
**Voles**					
Rv =	29.170 (5.23, 3.89)	−0.6133 [V_t−1_/R_t−1_] (3.39, 3.87)	−0.2178 [K_t−1_/V_t−1_] (1.01, 1.41)	+1.1199 Ta_t_ (1.82, 2.83)	0.0749
**Shrews**					
Rs =	172.551 (1.73, 3.25)	−3.9292 S_t−1_ (0.59, 1.90)	−4.3631 [K_t−1_/S_t−1_] (0.74, 1.41)		0.419
Rs =	420.940 (7.62, 3.25)	−4.5995 S_t−1_ (6.31, 2.14)	−5.2657 [K_t−1_/S_t−1_] (8.03, 2.49)	+22.5879 Ta_t_ (1.69, 5.57)	0.3094

Parameter values of selected PCGR models of kestrels (both periods), voles and shrews. A bias parameter was calculated as Σ (O_i_−P_i_)/n, where O_i_ is observed data, P_i_ is predicted data. Models showing closer values to 0 predicts better the data. Approximate 95% confidence intervals calculated with asymptotic approximation appear in parenthesis.

**Figure 4:**
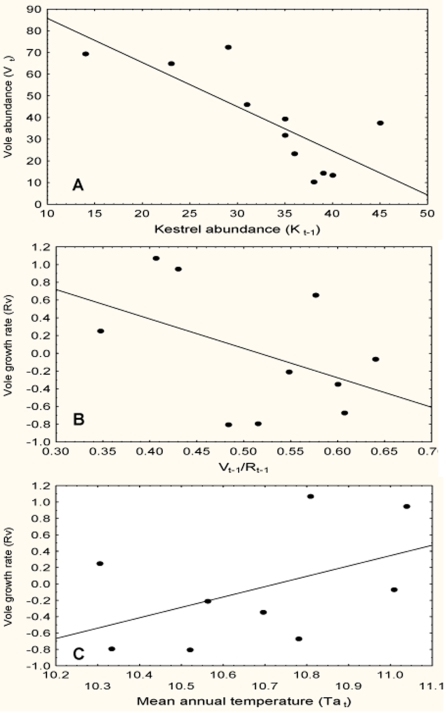
Factors affecting vole population dynamic. Linear relationship between common vole density and Eurasian kestrel density of the preceding year (A). Linear relationship between the per capita growth rate of common voles and the ratio of vole density to rainfall (B) and annual ambient temperature (C).

Vole results showed that the best approximating model for the data was a logistic model (model 27v) including both trophic terms arising from the density ratios of predator to prey (kestrels/voles) and prey to food resource (voles/rain) and a positive effect of mean annual temperature (Table 3). The second best model showed *ΔAICc*>6 for which reason it is a poorer candidate model with respect to the first. The model explaining the highest percentage of the variance follows a Lotka-Volterra formulation including four variables: vole and kestrel densities of the preceding year plus both climatic factors of rain and temperature. However, the *AICc* of this model differs by 15.21 units from the *AICc* of the best model. Hence, vole density in our study area seems to be affected by kestrel predation (Fig. 4A), food resources (rainfall, Fig. 4B) and air temperature (Fig. 4C). The abundance of kestrels of a given year predicts the abundance of voles for the next year (Fig. 4A), so that the higher the abundance of kestrels the lower the abundance of voles (*r* = −0.78, *F*_1,9_ = 14.01, *P* = 0.005).

**Table 3:**
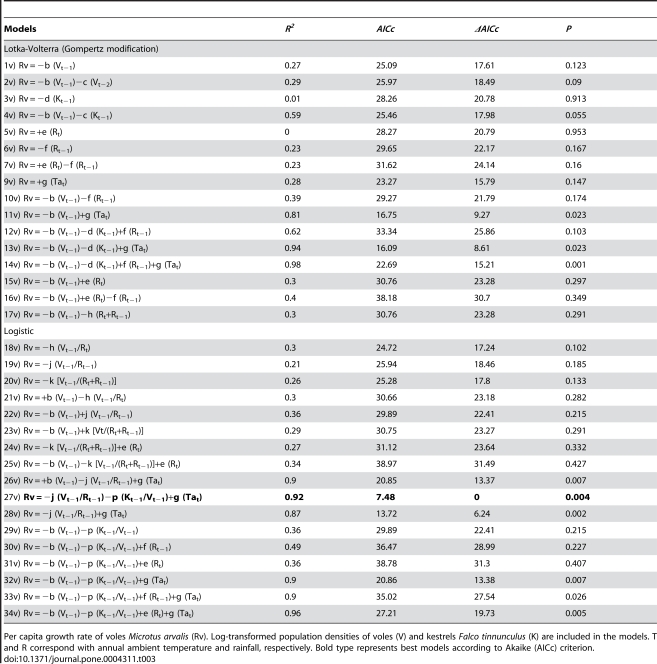
Selected vole population-dynamic models.

Per capita growth rate of voles *Microtus arvalis* (Rv). Log-transformed population densities of voles (V) and kestrels *Falco tinnunculus* (K) are included in the models. T and R correspond with annual ambient temperature and rainfall, respectively. Bold type represents best models according to Akaike (AICc) criterion.

**Figure 5:**
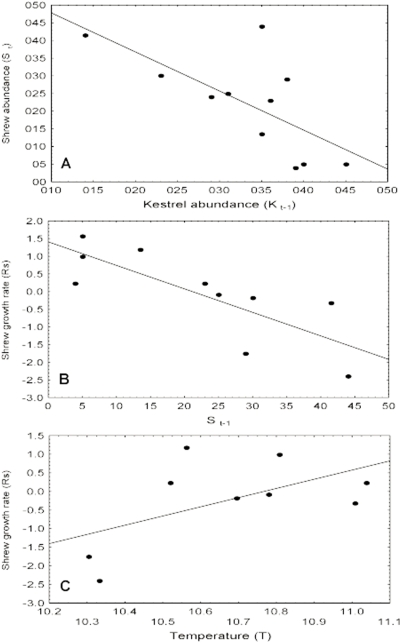
Factors affecting shrew population dynamic. Linear relationship between white-toothed shrew density and Eurasian kestrel density of the preceding year (A). Linear relationship between the per capita growth rate and one-year lagged density of shrews (B) and annual ambient temperature (C).

The most parsimonious model found for shrew PCGR (model 16s) described a logistic food web composed of the self-regulation term (shrew density of the preceding year) and the ratio of kestrel to shrew densities (Table 4). We found a second model (model 21s) that differs only by 1.08 units from the *AICc* of the best model, and can thus be considered as a candidate model. This model is similar to the first but includes temperature as an additive climatic force. Shrew growth rate increased when the density of shrews (density dependence) and kestrels of the preceding year was low (Fig. 5A,B) and when the mean annual temperature increased (Fig. 5C).

**Table 4:** Selected shrew population-dynamic models.

Models	*R^2^*	*AICc*	*ΔAICc*	*P*
Lotka-Volterra (Gompertz modification)				
1s) Rs = −b (S_t−1_)	0.5	32.65	4.4	0.029
2s) Rs = −b (S_t−1_)−c(S_t−2_)	0.59	35.36	7.11	0.069
3s) Rs = −d (K_t−1_)	0.07	38.94	10.69	0.458
4s) Rs = −b (S_t−1_)−c (K_t−1_)	0.7	33.51	5.26	0.014
5s) Rs = −b (S_t−1_)−c (K_t_)	0.5	38.61	10.36	0.085
6s) Rs = +c (R_t_)	0.17	36.52	8.27	0.123
7s) Rs = −d (R_t−1_)	0.24	36.92	8.67	0.15
8s) Rs = +c (R_t_)−d (R_t−1_)	0.44	39.77	11.52	0.127
9s) Rs = +c (R_t_+R_t−1_)	0.01	43.67	15.42	0.953
10s) Rs = +d (Ta_t_)	0.25	32.71	4.46	0.168
11s) Rs = −b (S_t−1_)−d (K_t−1_)+e (R_t_)	0.79	39.07	10.82	0.018
12s) Rs = −b (S_t−1_)−d (K_t−1_)+e (R_t−1_)	0.7	42.51	14.26	0.051
13s) Rs = −b (S_t−1_)−d (K_t−1_)+e (R_t_)+d (Ta_t_)	0.74	53.07	24.28	0.163
14s) Rs = −b (S_t−1_)−d (K_t−1_)+d (Ta_t_)	0.64	40.9	12.65	0.006
15s) Rs = −b (S_t−1_)+e (R_t_)	0.76	31.23	2.98	0.006
Logistic				
16s) Rs = −d (K_t−1_/S_t−1_)	0.3	36.11	7.86	0.101
**17**s) **Rs = −b (S_t−1_)−d (K_t−1_/S_t−1_)**	**0.82**	**28.25**	**0**	**0.002**
18s) Rs = −b (S_t−1_)−d (K_t−1_/S_t−1_)+e (R_t_)	0.87	34.04	5.79	0.004
19s) Rs = −b (S_t−1_)−d (K_t−1_/S_t−1_)+e (R_t−1_)	0.83	36.71	8.46	0.009
20s) Rs = −b (S_t−1_)−d (K_t−1_/S_t−1_)+e (R_t−1_)+d (Ta_t_)	0.91	43.77	15.52	0.023
21s) Rs = −b (S_t−1_)−d (K_t−1_/S_t−1_)+e (R_t_)+d (Ta_t_)	0.92	42.07	13.82	0.163
**22**s) **Rs = −b (S_t−1_)−d (K_t−1_/S_t−1_)+d (Ta_t_)**	**0.9**	**29.33**	**1.08**	**0.006**
23s) Rs = −b (S_t−1_)+b (V_t−1_/R_t−1_)	0.51	38.48	10.23	0.089
24s) Rs = +b (S_t−1_)−b [V_t−1_/(R_t_+R_t−1_)]+e (R_t_)	0.77	40.07	11.82	0.025
25s) Rs = +b (S_t−1_)−b (K_t−1_)−c (V_t−1_/R_t_)−d (Ta_t_)	0.72	54.02	25.77	0.197
26s) Rs = +b (S_t−1_)−b (K_t−1_)−c (V_t−1_/R_t_)+d (Ta_t−1_)	0.79	53.81	25.56	0.057
27s) Rs = +b (S_t−1_)−c (V_t−1_/R_t_)+d (Ta_t−1_)	0.8	38.71	10.46	0.017
28s) Rs = +b (S_t−1_)−c (V_t−1_/R_t_)−d (K_t−1_/S_t−1_)+d (Ta_t−1_)	0.91	43.87	15.62	0.024
29s) Rs = −c (V_t−1_/R_t_)−d (K_t−1_/S_t−1_)+d (Ta_t−1_)	0.77	21.97	8.72	0.046
30s) Rs = +b (S_t−1_)−c (V_t−1_/R_t_)−d (K_t−1_/S_t−1_)	0.83	36.71	8.46	0.009
31s) Rs = −c (V_t−1_/R_t_)−d (K_t−1_/S_t−1_)	0.72	33.09	4.84	0.012

Per capita growth rate of white-toothed shrews *Crocidura russula*. Log-transformed population densities of shrews (S) and kestrels *Falco tinnunculus* (K) are included in the models. T and R correspond with annual ambient temperature and rainfall, respectively. Bold type represents best models according to Akaike (AICc) criterion.

**Figure 6:**
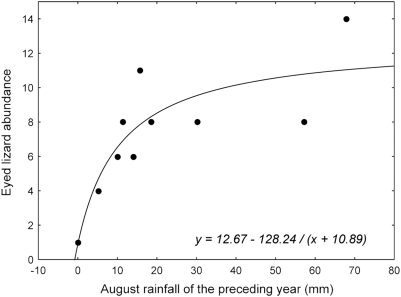
Lizard and August rainfall relationship. Hyperbolic relationship between annual abundance of eyed lizards and August precipitation of the preceding year.

Missing summer data from 1999 did not allow us to perform PCGR models for the eyed lizard with the methods employed here. Meteorological variables were not significantly correlated to lizard abundance (all *P*>0.11). By exploring in more detail the incidence of precipitation on lizard abundance variation, we found that August precipitation of the previous year positively affected lizard population size (*r* = 0.73, *F*_1,8_ = 8.93, *P* = 0.017). The response of lizard abundance to August precipitation of the preceding year was however better adjusted to a hyperbolic rather than to a linear function, since the former explained more variance (71.0%; *F*_1,8_ = 44.21, *P*<0.001, Fig. 6) than a linear function (52.7%). Lizard abundance was not significantly correlated to kestrel abundance of the preceding year (*r* = 0.49, *F*_1,8_ = 2.58, *P* = 0.147).

## Discussion

The apparent richness of prey species and the remarkable absence of nest sites for kestrels (trees, cliffs and buildings) prompted population numbers of this predator species to increase to habitat carrying capacity through the provision of nesting sites. Eurasian kestrels in our study area predate on the three species considered in the study: common voles, eyed lizards and white-toothed shrews, however, only common vole densities showed an effect on kestrel population rate of change. We first analysed kestrel population dynamics during a seven-year period in order to avoid the effect of nest-box management on kestrel numbers. The results obtained from these analyses coincided with those found when analysing the whole 11-year period studied. This lends more merit to our short time series. Nevertheless, the results did not allow us to select a specific model that defines kestrel growth rate. In any case, our models indicate that self-regulation and vole density seems to be important factors modulating kestrel population dynamics. The sum or a conjunct variable of the densities of the three prey-species included in the model would identify the most parsimonious PCGR-function for the population dynamics of a generalist predator. However, this was not the case, probably because the common vole is a major key species in this predator-prey system. Even when common voles represent 1.8% of the prey consumed and 7% of biomass in general, its consumption can increase drastically in years of high vole abundance with respect to the rest of the prey species (unpublished data), suggesting that it is a preferred prey species. Another explanation is the association between precipitation and vole density. Rain had a positive effect on vole densities but also had positive effects on other kestrel prey species, such as Orthoptera insects (field crickets, mole crickets and grasshoppers) that can fluctuate in a similar way to voles (see below).

The study site is located in a mountainous Mediterranean area with cold winters where the ground may be covered by snow from 22 to 51 days of the year and with warm and dry summers. Rainfall is a prime stimulus for increased primary productivity [42] and is particularly important in Mediterranean regions, where dry summers make the ecosystems, and vegetation in particular, strongly dependent on the rain fallen some months before [53]. Rainfall produced a significant increase in ephemeral (herb) cover and seed densities [54]–[56] and high rainfall years were associated with insect outbreaks [57], thus producing a significant increase in food availability for granivorous, folivorous and insectivorous small mammals [9], [19], [55], [57]. Vegetation growth increases with rainfall providing direct food sources for herbivorous species such as the common vole, but our results suggest that there may also be a more indirect effect, by increasing abundances of herbivorous invertebrates, thus increasing food resources for insectivorous eyed lizards and shrews. In addition, it is known that microhabitats of both small mammals and lizards are conformed by high vegetation cover [58]–[60] that provides good refuge against predators [43], [61], [62]. The number of days with snow covering the ground (one month on average) and days of frost in our study area is relatively high for a Mediterranean region. Warmer years, and particularly warmer winters, at high altitude prolong the growing season of plants by preventing or reducing the dormancy period which promotes an increase in vegetative growth [63]. These environmental aspects could explain the additive (temperature) and non-additive (precipitation) forces that modulate inter-annual fluctuations in the growth rate of voles, shrews and lizards in our study area.

The common vole is the studied rodent species showing the greatest variability in patterns of population dynamics. An analysis performed of 36 populations from Eastern Europe showed that 10% of them did not show clear periodicity in their inter-annual oscillations, and in the remaining populations the length of the dominant period (cycle) varied between 2 and 10 years [64]. However, in that study, many time-series data were not long enough to be conclusive. Studies in Poland, Czech Republic and Slovak Republic have shown that 62% of 29 common vole time-series analysed did not show density dependence and were not cyclic [65]. Four populations studied in western France showed, however, cyclic fluctuations [27]. Another population compilation by Turchin [3] with longer time-series from France, Poland and Russia showed a preponderance of first-order dynamics and only 25% of 20 populations reviewed showed second or higher order in density auto-regressions. Two of these populations from France (Brioux and Beauvoir) showed first-order dynamics when analysed by Turchin [3], while they showed second or third order when analysed by Lambin et al. [27], probably due to the use of different approaches: total abundances [3] or growth rate [27]. Our population showed no density-dependent structure and no regularity in inter-annual oscillations, as no significant models were found when including lagged densities of voles. It seems that in our case fluctuations in vole numbers are mainly constrained by exogenous influences such as temperature and rainfall that leave little room for the role of kestrel predation on vole dynamics. Otherwise a clear second-order inter-population process would have been found. Even so, we found that the model better explaining vole rate of change was a logistic model in which kestrel predation pressure is present together with the effect of rainfall and temperature.

Vole growth rate was negatively correlated with the ratio of kestrel to vole density. This shows that vole population grows the least when kestrels are abundant and suggests that kestrels could integrate an endogenous explanation (inter-population negative feedback) of vole dynamics, this being the effect observed when climatic factors are controlled for. This explains the asymmetrical interaction [49] between vole and kestrel densities. The effect of rain was incorporated in the consumer/resource ratio, signifying that rainfall constitutes what Royama [66] called a ‘lateral perturbation in population dynamics’ and implying that rainfall acts as an exogenous factor influencing a vole resource such as food (herb), as discussed above. This affects the carrying capacity (K), causing changes in the level of the equilibrium point of the population [67]. The effect of temperature is less clear. In this case, temperature shows an additive effect, also called vertical perturbation [66], suggesting that temperature can affect survival or reproduction, thus altering directly the PCGR. Warmer years can increase food and also refuge supply by boosting vegetative growth during winter, as previously noted.

Great white-toothed shrew is predated by kestrels in very low proportions (0.1%). In fact, shrew prey remains in kestrels nests are only found when shrew density peaks, such as in years 1997 or 2004 (unpublished data). This could explain why a non mutual effect has been found between both species. The two best models defining shrew PCGR (17s and 22s) show a kestrel effect (integrated in the consumer/resource ratio) in addition to a self-regulation effect. Shrew dynamics are explained by a first-order feedback structure determined by one-year lagged shrew densities and influenced by kestrel predation that is not translated, however, to a second-order structure. The other best model (22s) adds an additive influence of mean annual air temperature. Together with the potential effect of temperature indicated above, in the case of shrews, warmer years can benefit shrew population growth by providing longer seasons of insect activity, thus increasing carrying capacity of the habitat for shrews.

Although the eyed lizard represents an important prey species in the kestrel diet during the breeding season (19% of biomass), we did not find an effect of this species on kestrel PCGR. The real role of lizards in kestrel diet is lower as lizards are not predated during autumn, winter and early spring in our study area (unpublished data). The abundance of eyed lizards was described by a hyperbolic function associated with rainfall at the end of summer (August) of the preceding year. A positive effect of rainfall during the preceding summer has been observed in other lizard species from arid environments [46]. Precipitation can have a positive effect by increasing the number of invertebrates associated with vegetation such as grasshoppers, crickets and beetles and consequently promoting higher reserve accumulation to face the hibernation period which is longer at higher altitudes [68]. A second possibility is the positive effect that rainfall exerts by improving food conditions for incubation and hatching of late-season clutches that speeds up growth and increases juvenile survival [Haynes 1996 in 46]. In any case, there seems to be a threshold in the effect of summer precipitation, above which it has no further influence on lizard numbers. This could be due to a sampling error, because no more than 14 individuals (1997) of this territorial species could be captured in our trapping area [68]. Kestrel abundance did not apparently affect lizard density.

This study shows an analysis of preliminary 11-year data regarding population dynamics of a generalist predator and some of its prey species in a Mediterranean region. The most striking result of this study is the lack of second-order structure in the population dynamics of the three studied species. In the common vole we did not even find a first-order feedback or density dependence. The absence of this kind of dynamic could be due to the dominance of stochastic influence arising from climatic effects. In this sense, this study reports for the first time the effect of rainfall and ambient temperature on the population dynamics of the common vole. Veiga [69] also found an effect of autumn precipitation on the presence of common voles recorded in pellets of long-eared owls *Asio otus* in the same study area. Climatic variables modulated vole and shrew PCGRs in combination with kestrel density. Our results support expected from generalist predation, that is, a stabilization of prey populations as generalist predators prey on a particular species when this species is abundant. Predators may change to other prey when their primary prey becomes scarce, preventing outbreaks and crashes [34], [70] and/or promoting low-amplitude (SD of log-transformed densities) inter-annual cycles in temperate and southern Europe. This may primarily occur because generalist predators can behave in such a way that promotes a functional response that is destabilizing around the point of equilibrium [10]. Similarly, we found irregular fluctuations in time of vole and shrew population densities. In addition, inter-annual vole fluctuations in our study showed an amplitude of 0.28, which is notably low compared with other European populations [3].
